# How Well Do Raters Agree on the Development Stage of *Caenorhabditis elegans*?

**DOI:** 10.1371/journal.pone.0132365

**Published:** 2015-07-14

**Authors:** Annabel A. Ferguson, Richard A. Bilonick, Jeanine M. Buchanich, Gary M. Marsh, Alfred L. Fisher

**Affiliations:** 1 Department of Biostatistics, Graduate School of Public Health, University of Pittsburgh, Pittsburgh, Pennsylvania, United States of America; 2 Department of Ophthalmology, School of Medicine, University of Pittsburgh, Pittsburgh, Pennsylvania, United States of America; 3 Department of Orthodontics, School of Dental Medicine, University of Pittsburgh, Pittsburgh, Pennsylvania, United States of America; 4 Center for Occupational Biostatistics and Epidemiology and Department of Biostatistics, Graduate School of Public Health, University of Pittsburgh, Pittsburgh, Pennsylvania, United States of America; 5 Division of Geriatrics, Gerontology, and Palliative Medicine, Department of Medicine, UTHSCSA, San Antonio, Texas, United States of America; 6 Center for Healthy Aging, UTHSCSA, San Antonio, Texas, United States of America; 7 San Antonio GRECC, STVAHCS, San Antonio, Texas, United States of America; Inserm U869, FRANCE

## Abstract

The assessment of inter-rater reliability is a topic that is infrequently addressed in *Caenorhabditis elegans* research, despite the existence of sophisticated statistical methods and the strong interest in the field in obtaining reliable and accurate data. This study applies statistical modeling as a robust means of analyzing the performance of worm researchers measuring the stage of worm development in terms of the two independent factors that comprise “agreement”, which are (1) accuracy, representing trueness, a lack of systematic differences, or lack of bias, and (2) precision, representing reliability or the extent to which random differences are small. In our study, multiple raters assessed the same sample of worms to determine the developmental stage of each animal, and we collected data linking each scorer with their assessment for each worm. To describe the agreement of the raters, we developed a structural equation model with latent variables and thresholds, which assumes that all the raters are jointly scoring each worm. This common factor model separately quantifies the two aspects of agreement. The stage-specific thresholds examine accuracy and characterize the relative biases of each rater during the scoring process. The factor loadings for each rater examine the precision and characterizes the random error of the rater. Within our group, we found that the overall agreement was good, while certain adjustments in particular raters would have decreased systematic differences. Hence, the use of developmental stage as an experimental outcome can be both accurate and precise.

## Introduction

The ability to quickly, accurately, and precisely classify the stages of development for the nematode *Caenorhabditis elegans* is crucial for many aspects of worm research, including monitoring development, stock maintenance, crossing, and stage synchronization. In addition, the measurement of developmental stages within a population is useful in many studies. For example, mutations in genes, such as the *daf-2* insulin-like receptor pathway, alter normal development and lead to inappropriate arrest in the dauer stage [1] Further, a class of genes known as heterochronic genes result in abnormal development by disrupting the normal patterns of cell division and differentiation steps in specific stages [2]. Consequently research focused on these classes of genes have used visual assays to classify the stages of worm development and then to draw conclusions about both the individual genes involved in these pathways and gene-gene interactions between them. One common method for identifying and measuring the numbers of dauer stage animals within a population has been the use of sodium dodecyl sulfate (SDS) selection to kill non-dauer worms, and followed by recording the number of dauer and non-dauer larvae identified [3]. However, this method does not distinguish between other stages of development (L1, L2, L3, L4 and adult), and it is not able to identify partially-formed dauers, that show many but not necessarily all of the features for the dauer larva, in the count. A second approach is the use of a stage-specific reporter gene, such as the *col-19p*:*gfp* reporter which allows adult animals to be readily visualized by fluorescence [4]. But, this approach requires the prior identification of a gene showing stage-specific expression, and the ability to generate a reporter that exhibits both highly specific and readily visible fluorescent protein expression. A third approach is the use of imaging techniques and computer software, such as the WormSizer ImageJ plug-in, to measure worm size in an objective manner [5]. While the use of measurements can demonstrate differences in the developmental rate or body size of mutants, determining if the differences reflect alterations in specific growth and/or developmental processes could be more challenging using this approach alone. Consequently, visual assessment is an attractive way to obtain detailed information about larval development within a population both quickly and without the need for specialized strains or equipment.

However, a drawback to this method is the potential for variability arising from differences in how individual raters perceive each stage of development. The issue of potential disagreement between raters on the developmental stage of the same worm, also known as inter-rater agreement, has been addressed and analyzed in other fields such as psychology and medicine [6, 7]. However, there is little mention of formal statistical modeling of inter-rater agreement in *C*. *elegans* publications using measurements of developmental stage. Given both concerns about data reproducibility and the interest in inter-rater agreement in other fields, we sought to apply statistical analysis to this basic aspect of worm research to explore the operating parameters of visual scoring.

To address the variability between different researchers evaluating stage of development by eye, it is necessary to generate a dataset consisting of the developmental stage assigned to each animal within a sample of worms by multiple raters. The developmental stage assessment is an outcome consisting of an ordered categorical variable, given that the different stages are recorded as discrete qualities, with a natural order based on the progression between L1, L2, dauer, L3, L4, and adult. A computationally simple and commonly used statistical method for describing inter-rater agreement utilizing this type of ordered categorical data is through the calculation of the kappa statistic [6, 8]. This statistic is a single value that is interpreted as the amount of agreement exceeding that which is expected by chance alone. While the kappa statistic is a popular type of analysis due to the ease of computation, and the apparent simplicity of interpretation, there are also a number of criticisms that make use of the kappa statistic problematic. Important problems with the kappa statistic are two-fold. First, the kappa statistic sometimes may give rise to paradoxical or misleading results with certain arrangements of data, and second, it combines the two independent components of agreement (accuracy and precision) into one parameter which precludes a clear description of how the raters disagree [9–12]. Latent variable modeling provides a more insightful method for analyzing inter-rater agreement for ordered categorical measurements [9]. This approach has the advantage of providing information concerning the accuracy and precision of each rater. However, it should be noted that in most applications, the true developmental stage of each worm is unknown, so that only relative accuracy can be assessed.

The basic concept of agreement among measurements is relevant whether the measurements are of a discrete, ordinal nature or whether they are continuous and quantitative. A simple but limited approach to assessing agreement between two continuous and quantitative measurements consists of making a plot of the pairwise differences versus the corresponding pairwise averages. In the medical literature, this approach was championed by Bland and Altman and the plots are often referred to as Bland-Altman plots [13]. If the pairwise differences are normally distributed, then it is easy to compute the “limits of agreement” that demarcate where 95% of the differences will tend to fall. If the limits of agreement are very narrow with regards to the outcome, then for all intents and purposes, the measurements made by the two methods are interchangeable. When this is not the case, then it is necessary to characterize what part of the difference is systematic and what part is random. This is important because the systematic error can be removed by determining the appropriate calibration equation. Unfortunately, when the limits of agreement are too large to be considered negligible, the limitations of this approach are readily apparent because with only two measurements, it is impossible to apportion the error into systematic and random parts. As Bland and Altman (and many others) have pointed out, the usefulness of conducting a measurement error study of two methods without repeats of at least one of the measurements is questionable [13]. To have a useful study, at least one of the methods must be repeated (Bland and Altman suggested that both methods be repeated) or if neither method is repeated, at least three methods must be simultaneously compared.

Because of the discrete nature of ordinal measurements, Bland-Altman plots are of limited usefulness for studying agreement among the raters of worm development stages. However, the relevance and importance of apportioning the error into its systematic and random components persists. This knowledge will guide efforts to remove the tendency for raters to disagree. For example, the prescription for removing disagreement among raters will differ if there is little systematic error but high imprecision (tendency for large random errors) versus the reverse situation.

To overcome the limitations of the kappa statistic and Bland-Altman plots and to provide an analysis of the precision and accuracy of the individual raters, we describe how the common factor model with thresholds can be applied to the ratings of worm developmental stage performed by members of a *C*. *elegans* research group. We then further use the model to perform a detailed evaluation the precision and accuracy of the individual raters as a means of studying the reliability and limitations of visual scoring of worm development in a research setting. Our methods and findings are relevant for understanding the performance of visual scoring of development as an experimental outcome, and provide a means to evaluate the assessment of other phenotypes that depend on rater performance in *C*. *elegans* research.

## Results

### Development of the common factor model

**Fig 1:**
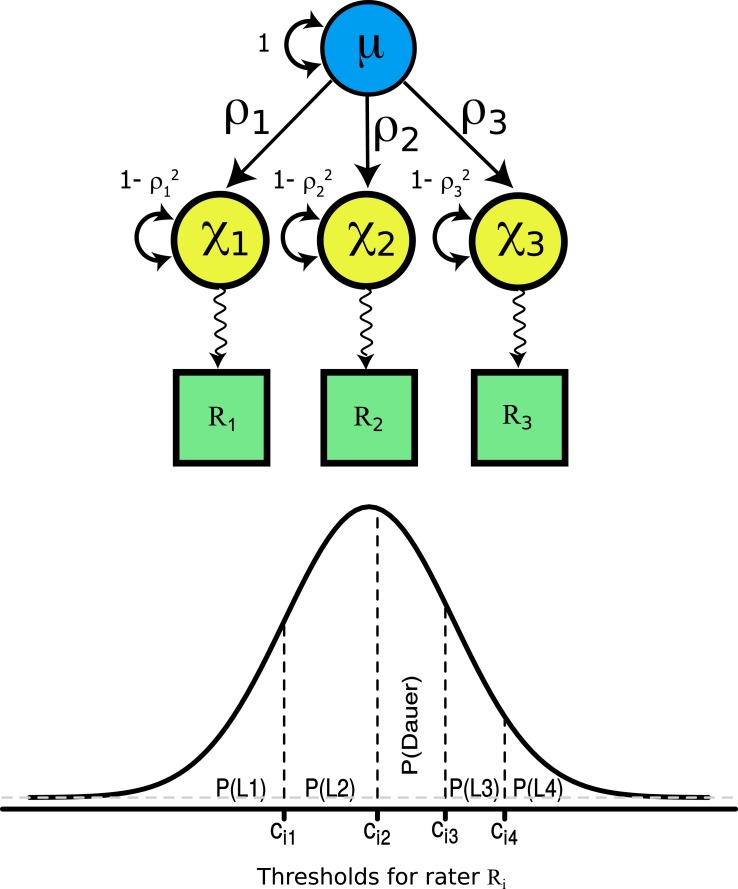
A common factor ordinal model to analyze rater agreement. This model describes the ordinal measurements (R_1_, R_2_, and R_3_) made by three raters (1, 2, and 3), which are observed (manifest) variables denoted by squares. These variables are related to the variables μ and χ, which are latent, meaning that they are not directly observable, but are included in the model since they underlie the actual observable values. The latent variable μ corresponds to the true worm stage but on a continuous scale. The variable μ is defined as being normally distributed with a mean of zero and a standard deviation of one. This standard deviation is represented by the curved arrow showing the value one (“1”) that is adjacent to μ. Each rater judges the stage of worm development on his or her own continuous scale, shown as the latent variables χ_1_, χ_2_, and χ_3_ in the model. Each rater’s unknown continuous scale is a linear function of μ as indicated by the single arrow paths pointing from μ to each χ. The slopes (path coefficients) for these linear functions are denoted by ρ_1_, ρ_2_, and ρ_3_ and the intercepts are equal to zero. These functions result in χ_1_, χ_2_, and χ_3_ having a residual standard deviation of 1-ρ_1_^2^, 1-ρ_2_^2^, and 1-ρ_3_^2^, respectively, which are denoted by the labelled curved arrow beside each variable. The directed path from each rater’s continuous scale, χ, and the observed ordinal measurement, R, is nonlinear as denoted by the sinusoidal path. The nonlinear relationship can be described as a threshold model where the thresholds (c_i1_, c_i2_, c_i3_, and c_i4_) for rater i control the marginal probability of each observed ordinal measurement (denoted by P(L1), P(L2), P(dauer), P(L3), and P(L4)) under the assumption that each rater’s continuous judgment is normally distributed with mean of zero and a standard deviation of one.

The common factor model approach assumes that all of the raters are jointly rating the same worm, and hence this model is applicable to the experimental design used in this work. The common factor model permits (assuming there are three or more raters and multiple worms) the decomposition of the rater differences into the part due to systematic error and the part due to random error. This decomposition is indirect in the sense that the scores are discrete ordinal categories, i.e., the L1, L2, dauer, L3, or L4 larval stages, and an underlying continuous scale must be assumed for the purposes of the model. Fig 1 illustrates the common factor model for an example involving three raters. The common factor is envisioned as the true worm stage but on a continuous scale, which is denoted by μ. Each rater judges the stage on his or her own continuous scale, shown as χ_1_, χ_2_, and χ_3_ in the model. Both variables μ and χ are latent, meaning that they are not directly observable, but are included in the model since they underlie the actual observable values. The true stage of worm development, μ, is assumed to be normally distributed with a mean of zero and a standard deviation of one. This standard deviation is denoted by the curved arrow adjacent to μ in Fig 1. Each rater then assesses worm development using their individual rating scale, denoted by χ_1_, χ_2_, or χ_3_. The variable χ_1_, χ_2_, or χ_3_ is related to μ via a linear function with an intercept of zero and a slope of ρ_1_, ρ_2_, and ρ_3_, respectively. This results in χ having a standard deviation of 1-ρ^2^, which is denoted by the curved arrows. The discrete ordinal ratings for an individual worm that are assigned by a rater are derived from each rater’s underlying latent continuous scale by applying the rater’s thresholds, which lie along the normal distribution. Because there are five categories, there will be four thresholds between them. The stages of development fall in regions along a standard normal distribution, and values for these threshold cutoffs are estimated from the observed ratings.

### Summary of rater data

**Fig 2:**
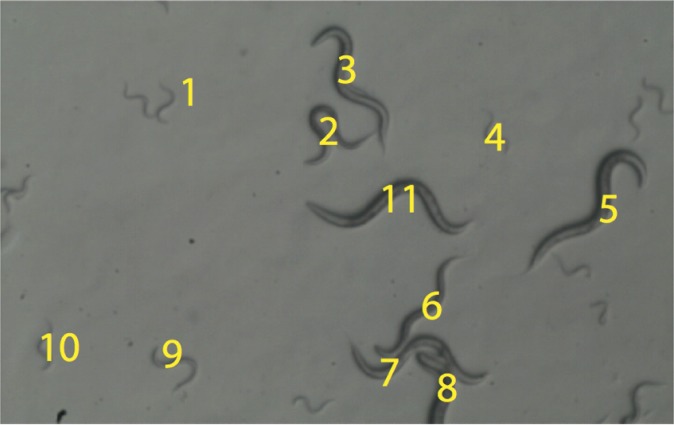
Sample still image of the sample of worms used for scoring by the raters. Each rater was assigned the same sample of worms to score for developmental stage. The worms were shown in both a 40X magnification image (illustrated) as well as a short video recording of each animal. Each worm was identified by a number to facilitate each rater evaluating identical animals in the same order.

To use the common model to compare inter-rater agreement, we asked seven different individuals, who had a range of experience in *C*. *elegans* research, to score 60 worms with regards to developmental stage (Table 1). To ensure that all raters scored the same worms, as required by the assumptions of the model, we used files consisting of both still and video images of each worm, and the individuals were asked to score each animal with regards to the stage of development (L1, L2, dauer, L3, or L4) (Fig 2). Review of the ratings showed that 42% of the time there was complete agreement among the raters, 35% of the time only a single rater differed from the group by a single stage, 12% of the time multiple raters differed with a split between them of a single stage, and 12% of the time multiple raters differed with a split of two stages (S1 Table). We then determined the frequency with which each rater scored the animals into each developmental stage. For the L1 stage, the highest and lowest frequencies were 0.36 and 0.20, for L2 these were 0.13 and 0.20, 0.10 and 0.23 for dauer, 0.08 and 0.20 for L3, and 0.18 and 0.30 for L4 (Observed column in Table 2). Hence, the largest discrepancy in scoring was present for the L1 stage, and among raters, individuals 1 and 4 showed the largest discrepancy. Additionally, raters 3, 4, 5, and 7 tended to place a larger fraction of worms in the extreme larval stages (L1 or L4) compared to the other individuals.

**Table 1:** Description of raters.

Rater	Years of Experience
1	10
2	5
3	1
4	2
5	<1
6	2
7	<1

Rater numbering with the number of years of *C*. *elegans* research experience.

**Table 2:** Expected proportions of larval stages for each rater based on model, and observed proportions.

Rater	L1		L2		Dauer		L3		L4	
	exp.	obs.	exp.	obs.	exp.	obs.	exp.	obs.	exp.	obs.
1	0.185	0.20	0.249	0.20	0.154	0.20	0.123	0.10	0.288	0.30
2	0.255	0.27	0.189	0.13	0.161	0.23	0.152	0.10	0.244	0.27
3	0.273	0.28	0.229	0.18	0.080	0.15	0.181	0.12	0.236	0.27
4	0.379	0.36	0.158	0.17	0.055	0.10	0.150	0.08	0.258	0.29
5	0.295	0.30	0.191	0.13	0.068	0.15	0.220	0.15	0.226	0.27
6	0.276	0.28	0.191	0.15	0.131	0.18	0.226	0.20	0.177	0.18
7	0.234	0.25	0.241	0.18	0.089	0.15	0.171	0.13	0.265	0.28
Average	0.270	0.28	0.210	0.16	0.110	0.17	0.170	0.13	0.240	0.27

Proportions of larval stages for each rater, as predicted by the model, and observed values.

**Fig 3:**
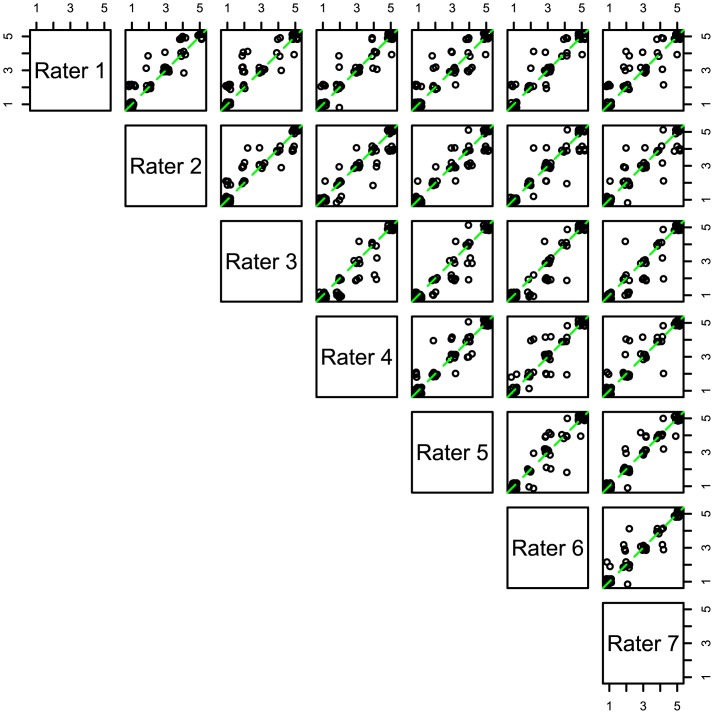
Head-to-head comparison of ratings from pairs of observers. Each of the 60 animal developmental stage ratings from a pair of reviewers is compared via the use of a pair-wise scatter plot matrix. The axis showing numbers 1 through 5 represents the animal stage with 1 representing L1, 2 representing L2, 3 representing dauer, 4 representing L3, and 5 representing L4. The green line represents perfect agreement between the two observers, and points along this line represent animals that are scored similarly by each observer. In contrast points either above or below the line represent disagreement between the raters. The ordinal values are slightly “jittered” to make it easier to discern the varying density of the ratings.

The discrepancy between individual raters can also be seen using pairwise error plots showing the results of one rater on the x-axis versus another rater on the y-axis (Fig 3). We used jittering to add a small amount of noise to the horizontal and vertical coordinates for the points to facilitate the visualization of data points that would otherwise be overlapping. In these graphs, any points falling away from the diagonal line correspond to discrepancies between the two raters, whereas points falling along the diagonal line demonstrate agreement. The pairwise plots show many points along the diagonal indicating agreement. No discrepancies were greater than two stages with most differences being just one stage (e.g., L1 larva versus L2) as opposed to two stages. The points tend to align best for the extreme stage (L1 and L4), whereas most of the discrepancies appear in the middle stages (L2, L3, and dauer) (Fig 3). These plots also demonstrate that for each pair of raters, there is variability regarding whether the disagreements fall above or below the diagonal line of agreement. For the pairs of raters where most of the discordant points lie either above or below the diagonal, this is suggestive of a directional bias of one rater compared to the other. For instance, in the plot comparing rater 2 versus rater 5 (Fig 3), the points fall roughly equally above or below the diagonal line, while in the plot comparing rater 3 versus rater 7, most of the discordant points lie below the diagonal line, suggesting a tendency of rater 3 to score the animals in later developmental stages compared to rater 7. Visualizing the data in these pairwise plots can suggest a bias or directionality to the disagreements as discussed above, and also can suggest random differences. The problem is that these plots cannot indicate how much of the observed difference is due to systematic error and how much is due to random error. In order to fully describe these two types of error among the seven raters, it is necessary to model the data, as described below.

### Fitting the data to the common factor model with thresholds

To further explore inter-rater agreement in terms of the bias and random error of the raters, we fit a common factor model similar to that shown in Fig 1 to the ordinal measurements, but with the seven raters instead of the three raters depicted. The parameters in this model are the standardized factor loadings ρ_1_ through ρ_7_, as well as four threshold values for each rater, c_i1_ through c_i4_. In this model, the factor loadings can be interpreted as the correlation between each rater’s stage assignments and the true developmental stage of the animals since all of the latent variable μ, which represents the true developmental stage of the animal has been standardized to have a mean of zero and a standard deviation of one. Further, the use of a standardized latent variable constrains the residual error standard deviations σ_*i*_ to be equal to $\sqrt{1-\rho^{2}}$. The thresholds can be interpreted as dividing the continuous latent scales into the ordered categories representing each developmental stage. In this model, there are seven factor loadings, one for each rater, and 28 (4 × 7) thresholds for a total of 35 parameters to be estimated.

### Factor loading estimates

We estimated each of the parameters including the factor loadings along with their corresponding 95% confidence intervals and the results are shown in Table 3. The residual error standard deviations that describe the rater imprecision were computed using the factor loadings using the formula in the previous section. Because of the inverse relationship between the factor loadings and the residual error standard deviations, a higher factor loading value results in a lower residual error standard deviation. The residual error standard deviation describes the raters imprecision while, conversely the factor loading describes the rater’s precision.

**Table 3:** Factor loading estimates ρ for the one factor ordinal model with seven raters.

		95% Confidence Interval Bounds for ρ					
Rater	Estimate of ρ	Lower	Upper	Estimate of ρ^2^	Residual Error SD $\sqrt{1-\rho^{2}}$	Scale Adjusted Residual Error SD $\frac{\sqrt{1-\rho^{2}}}{\rho }$	$\sqrt{2}$ Residual Error SD $\sqrt{2-2\rho^{3}}$
1	0.982	0.971	0.989	0.964	0.189	0.192	0.267
2	0.989	0.986	0.992	0.978	0.148	0.150	0.209
3	0.995	0.990	0.996	0.990	0.100	0.101	0.141
4	0.997	0.994	NA^1^	0.994	0.077	0.077	0.109
5	0.999	0.999	0.999	0.998	0.045	0.045	0.063
6	0.988	0.979	0.990	0.976	0.154	0.156	0.218
7	0.991	NA	0.993	0.982	0.134	0.135	0.189

Factor loading parameter estimates for the one-factor ordinal model, with the 95% confidence intervals, and the computed residual error for each rater.

^1^NA indicates instances where the computational algorithm could not obtain a lower bound or an upper bound for the likelihood-based confidence interval.

**Fig 4:**
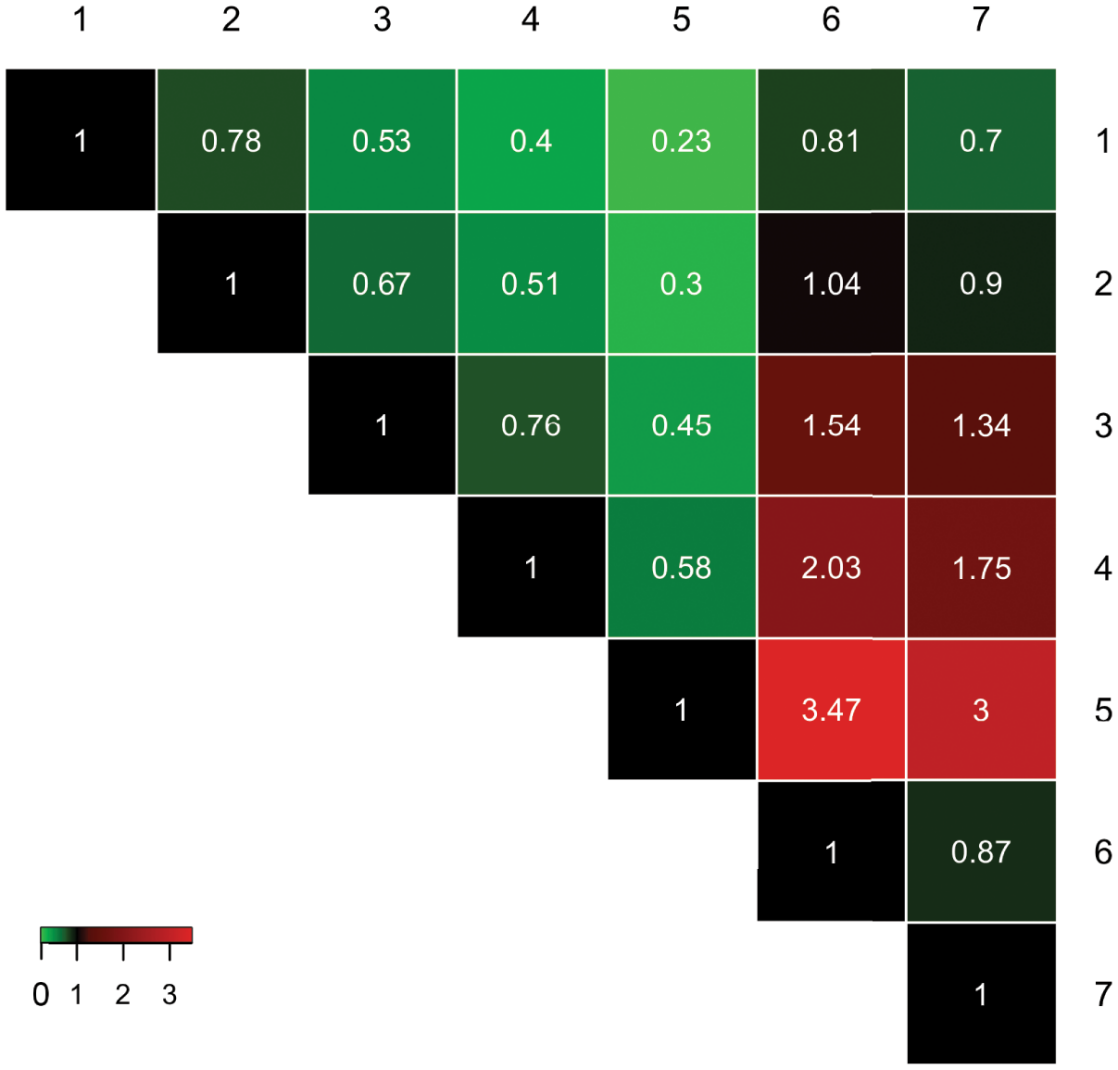
Heat map showing the pairwise ratio of the residual error estimates for all raters. The residual error estimate for the rater indicated in each row was divided by the rater in each column, and then was displayed as a heat map to highlight similarities and differences between raters. Each ratio is shown as the number inside of the colored box. The brightness of the color indicates relative strength of difference between raters, with red representing a ratio greater than one and green representing a ratio less than one.

At the same time, the factor loading describes the latent measurement scale for each rater, i.e., the size of the measurement unit. That is, differences in *ρ*_*i*_ among the raters imply different scales for the raters. On the latent scale, the marginal distribution for each rater has a standard deviation of one and a mean of zero. As a result, the residual error standard deviation $\sqrt{1-\rho^{2}}$ represents the uncertainty in the rater's latent continuous measurement conditional on the true latent value ρ and thus describes the imprecision, or lack of repeatability, of the rater's measurement process. The larger the residual error standard deviation or imprecision, the more likely that repeated measurements of an animal would fall into different ordinal categories. In order to compare raters in terms of imprecision, the differences in the raters’ scales (*ρ*_*i*_) must be taken into account. This is easily accomplished simply by dividing the residual error standard deviation by the corresponding factor loading:

$$\frac{\sqrt{1-\rho_{i}^{2}}}{\rho_{i}}\;=\;\sqrt{\frac{1}{\rho_{i}^{2}}-1}$$

and is referred to as the “scale-adjusted imprecision.” As the factor loading approaches 0, the scale-adjusted imprecision increases without bound. When the factor loading equals 1, the scale-adjusted imprecision equals 0 indicating perfect precision. As shown in Table 3, the raters in this study had scale-adjusted imprecision standard deviations that ranged from 0.045 to 0.192, with rater 5 being the most precise and rater 1 being the least precise. Overall, the order of rater precision (best to worst), based on lowest to highest residual error values is rater 5, 4, 3, 7, 2, 6, and then 1, which correspond to residual error values of 0.045, 0.077, 0.101, 0.135, 0.15, 0.156, 0.192, respectively. To facilitate comparison of the residual errors between raters, Fig 4 and Table 4 show the ratio of the residue error estimates between pairs of raters (columns divided by rows). The rater who stands out is rater 5, who has a considerably lower residual error estimate (as much as three times lower than other raters). Raters 1 and 2 have the highest residual error rates, and these differ the most with raters 3, 4, and 5. The magnitude of these error rates are discussed in more detail below.

**Table 4:** Ratios of residual standard errors for each rater pair.

Rater	1	2	3	4	5	6	7
**1**	1.00	0.78	0.53	0.41	0.24	0.81	0.71
**2**		1.00	0.68	0.52	0.30	1.04	0.91
**3**			1.00	0.77	0.45	1.54	1.34
**4**				1.00	0.58	2.00	1.74
**5**					1.00	3.42	2.98
**6**						1.00	0.87
**7**							1.00

Ratios of the residual standard errors, displaying the ratio of column divided by row value of the residual error for each rater, as calculated in Table 2

### Threshold estimates

**Fig 5:**
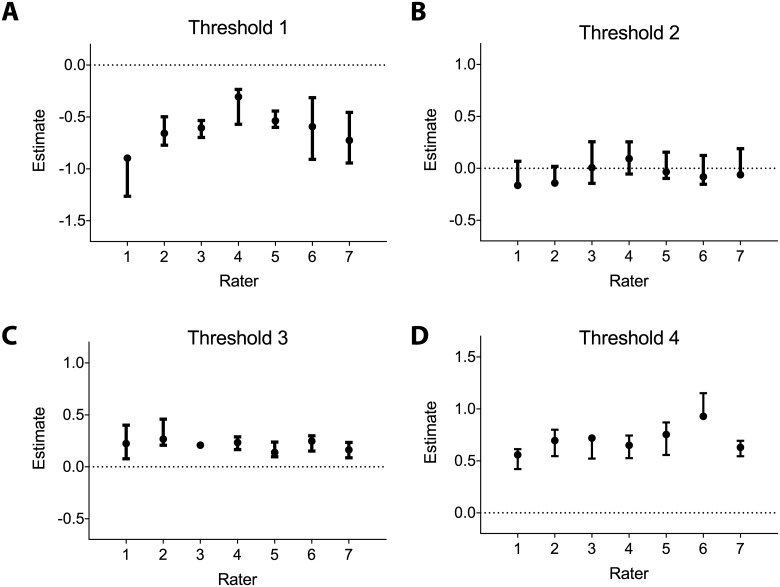
Rater-specific thresholds estimated using the common factor model. The thresholds classify the worms into the L1, L2, dauer, L3, or L4 stages. Each stages represents an abstract concept encompassing size, morphologic, and behavioral features of the worm that can be perceived by a rater relative to each threshold. Threshold 1 (A) separates the L1 and L2 categories, threshold 2 (B) separates the L2 and dauer categories, threshold 3 (C) separates the dauer and L3 categories, and threshold 4 (D) separates the L3 and L4 categories.

**Fig 6:**
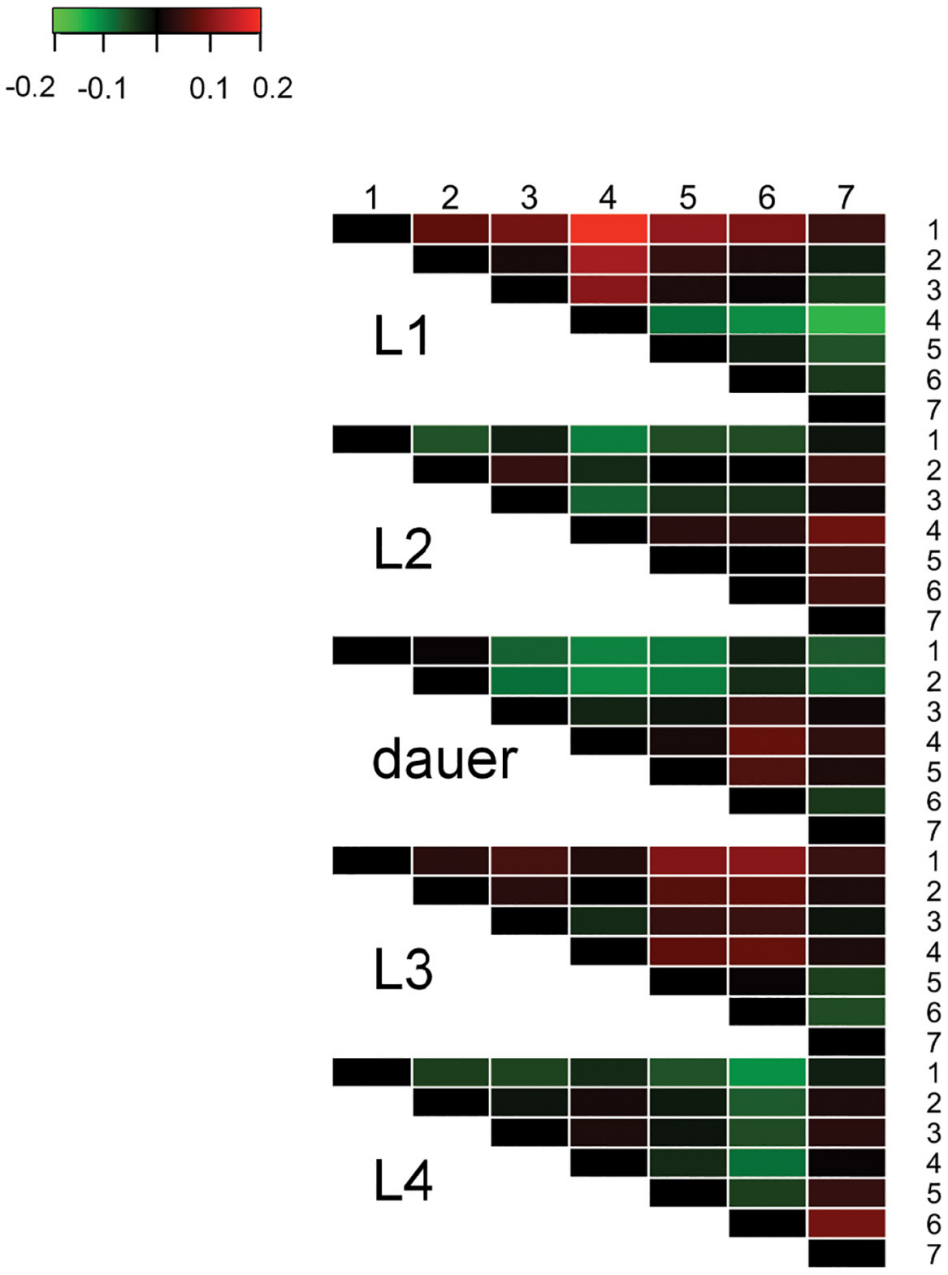
Heat map showing differences between raters for the predicted proportion of worms assigned to each stage of development. The brightness of the color indicates relative strength of difference between raters, with red as positive and green as negative. Result are shown as column minus row for each rater 1 through 7.

The factor loading estimates indicate how closely each rater’s assessment of developmental stage for a worm correlates with its true developmental stage and represents only random error, providing a description as to how consistently or reliably an individual rater classifies each worm. By contrast, the thresholds represent systematic differences among the raters in the location and width defining the ordinal stages on the latent continuous scale. In this model, threshold 1 divides the L1 stage from stage L2 stage on the latent continuous scale. Threshold 2 divides L2 from dauer, and so forth. The estimated thresholds based on the fitted model are shown in Table 5 and graphed in Fig 5. Based on these estimated thresholds, it is possible to determine the proportion of worms a particular rater would be expected to classify into a particular stage of development providing an alternative way of comparing the performance of the raters. Each proportion is simply the area under the unit-standard normal curve corresponding to the particular stage. The expected proportions of each stage of development for each rater are shown in Table 2 (Expected column). Comparing the expected proportions from rater to rater gives an indication of where particular raters systematically differ in how they classify worms. Fig 6 shows a visual heatmap display of the differences in expected proportion between raters (columns minus rows), with red indicating a positive difference and green indicating negative, and the brighter the color, the greater the difference. This display provides a means of quickly assessing the rater or raters that stand out as being considerably different from the others in the scoring of a particular stage of development. In our group data, for the L1 stage, rater 4 and rater 1 stand out as showing the highest and lowest proportion of animals being assigned to this stage, respectively. For the L2 stage, all raters are relatively similar, for the dauer stage, rater 2 and 4 have a relatively large difference in animals assigned to this stage, and for the L3 and L4 stages, rater 6 stands out as assigning considerably more animals to the L3 stage and correspondingly fewer to the L4 stage.

**Table 5:** Expected and observed threshold values for each rater.

	Threshold							
Rater	1		2		3		4	
	exp.	obs.	exp.	obs.	exp.	obs.	exp.	obs.
1	-0.897	-0.84	-0.165	-0.25	0.223	0.25	0.558	0.52
2	-0.658	-0.61	-0.142	-0.25	0.266	0.33	0.695	0.61
3	-0.605	-0.58	0.005	-0.10	0.208	0.28	0.718	0.61
4	-0.307	-0.36	0.092	0.08	0.233	0.33	0.650	0.55
5	-0.538	-0.52	-0.035	-0.18	0.137	0.20	0.753	0.61
6	-0.594	-0.58	-0.083	-0.18	0.247	0.28	0.928	0.88
7	-0.725	-0.67	-0.062	-0.18	0.162	0.20	0.629	0.55

Threshold values for each rater, predicted by the fitted model (exp.) and calculated from observed proportions of each stage (obs.)

### Assessing the relevance of rater differences

While the estimated factors discussed above provide a means of comparing different aspects of how each rater in a group scores worm developmental stage, it is important to consider whether these differences make a notable change in the experimental data collected by each rater. Specifically, one would want to know how much imprecision or bias may be shown by a rater without compromising the ability to produce reliable measurements of stages of worm development. To make this determination, one should consider both the residual error of each rater as well as the threshold estimates for each stage of development. If a rater were to make two independent, repeated measurements of the same worm, the standard deviation of the expected difference in the continuous latent measurements would be about 1.41 times the residual error standard deviation. These values would be for raters 1 through 7, 0.27, 0.21, 0.14, 0.11, 0.06, 0.22 and 0.19, respectively. These values may then be compared to the differences between the thresholds for a given rater. The smaller the threshold differences are compared to $\sqrt{2-{2\rho }^{2}}$, then the more likely imprecision is to result in repeated judgments that produce differences in the worm's developmental stage. This propensity varied depending on rater and stages. However, we found that for all of the raters, the threshold widths tended to be large compared to $\sqrt{2-{2\rho }^{2}}$, which suggests that rater bias plays a larger role in discrepancies in scoring than does imprecision. But, there are a few instances when threshold width was similar to $\sqrt{2-{2\rho }^{2}}$, such as the interval between threshold 2 and threshold 3 for rater 4 (Table 5). In these circumstances imprecision can play a larger role in the observed differences than seen elsewhere.

To investigate the impact of rater bias, it is important to consider the differences between the raters’ estimated proportion of developmental stage. For the L1 stage rater 4 is approximately 100% higher than rater 1, meaning that rater 4 classifies worms in the L1 stage twice as often as rater 1. For the dauer stage, the proportion of rater 2 is almost 300% that of rater 4. For the L3 stage, rater 6 is 184% of the proportion of rater 1. And, for the L4 stage the proportion of rater 1 is 163% that of rater 6. These differences between raters could translate to unwanted differences in data generated by these raters. However, even these differences result in modest differences between the raters. For instance, despite a three-fold difference in animals assigned to the dauer stage between raters 2 and 4, these raters agree 75% of the time with agreement dropping to 43% for dauers and being 85% for the non-dauer stages. Further, it is important to note that these examples represent the extremes within the group so there is in general more agreement than disagreement among the ratings. Additionally, even these rater pairs might show better agreement in a different experimental design where the majority of animals would be expected to fall in a specific developmental stage, but these differences are relevant in experiments using a mixed stage population containing fairly small numbers of dauers.

### Evaluating model fit

**Fig 7:**
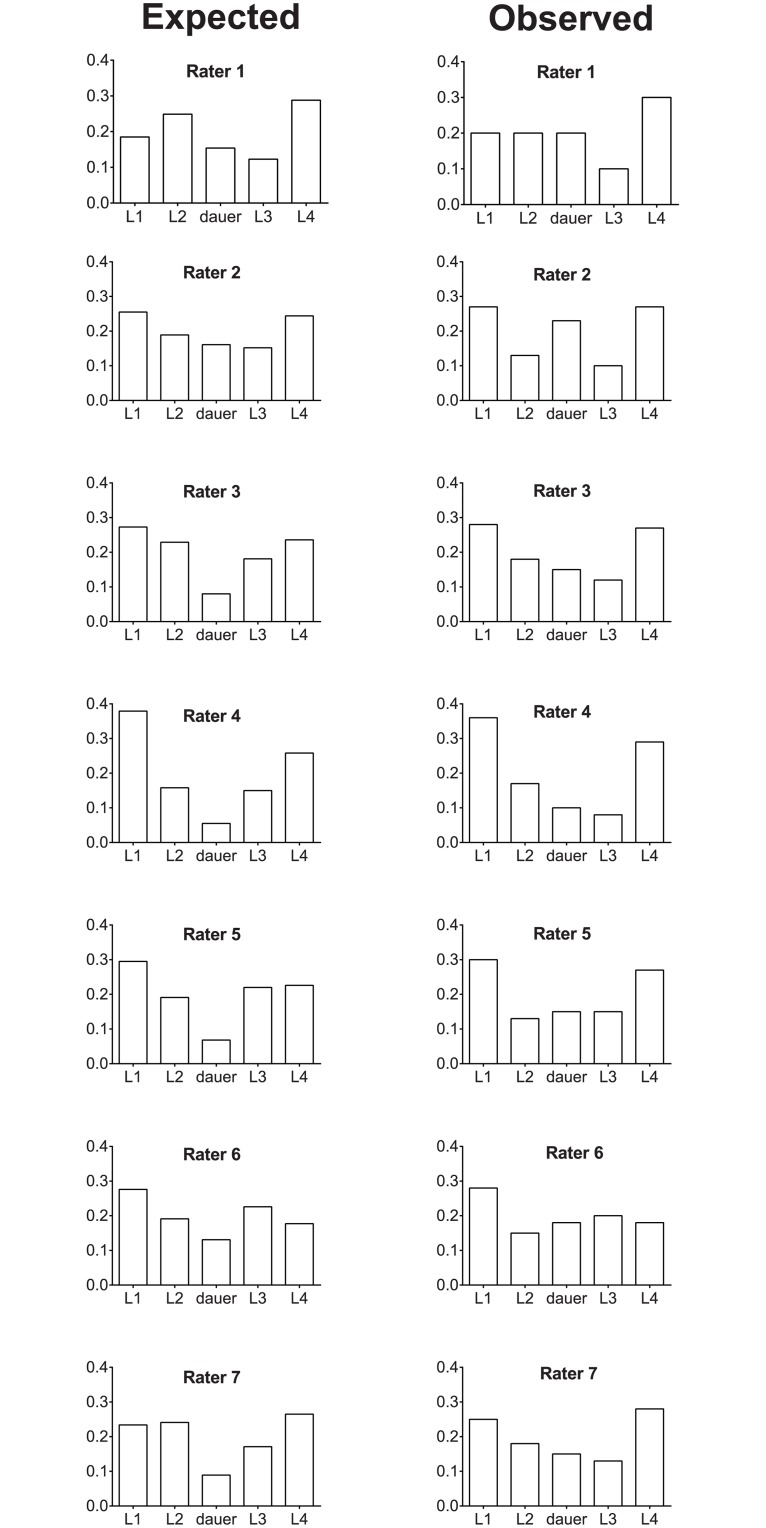
Comparison of the common factor model with rater behavior. Shown are bar-graphs depicting the percentages predicted for the assignment of animals to each stage by individual reviewers from the estimated common factor model (left column), and the observed percentage of animals assigned to each of the developmental stages for the raters (right column).

To examine how well the model fits the collected data, we used the threshold estimates to calculate the proportion of worms in each larval stage that is predicted by the model for each rater (Table 2). These proportions were calculated by taking the area under the standard normal distribution between each of the thresholds (for L1, this was the area under the curve from negative infinity to threshold 1, for L2 between threshold 1 and 2, for dauer between threshold 2 and 3, for L3 between 3 and 4, and for L4 from threshold 4 to infinity). We then compared the observed values to those predicted by the model (Table 2 and Fig 7). The observed and expected patterns from rater to rater appear roughly similar in shape, with most raters having a larger proportion of animals assigned to the extreme categories of L1 or L4 larval stage, with only slight variations being seen from observed ratios to the predicted ratio. In addition, model fit was assessed by comparing threshold estimates predicted by the model to the observed thresholds (Table 5), and similarly we observed good concordance between the calculated and observed values.

## Discussion

The aims of this study were to design and carry out an experiment for measuring inter-rater agreement among *C*. *elegans* researchers, to evaluate these data using a common factor latent variable model with thresholds, to draw conclusions about relative accuracy and precision among a group of raters, and to generate a protocol for measuring inter-rater agreement that other labs can follow. The major findings were that: 1) it is possible to quickly obtain data from multiple raters assessing the same sample of worms for five stages of development; 2) that visual assessment of pairwise error plots reveals some degree of differences among the seven raters, especially for the L2, dauer and L3 stages; and 3) that in the one factor model, raters tend to have high reliability (precision) but there are larger differences between raters in the threshold values for the stage boundaries (accuracy).

### Measuring inter-rater agreement in *C*. *elegans* researchers using visual assessment

The goal of our experimental design was to minimize any sources of variability in the animals used for scoring that would then be confounded by rater error. To ensure that each rater was making an assessment under the same visual conditions, a video recording of a magnified population of worms was used instead of having the raters look at live worms under a microscope. This both presented an identical animal for scoring to each rater, and enabled each worm to be given a unique identifier to eliminate the possibility that raters were assessing a different worm, or inadvertently scored the same worm twice. One criticism of this design is that by using a pre-recorded image, this could take away certain aspects of a visual assessment a rater may use when looking under a microscope, such as adjusting the amount zoom and focus. These adjustments may contribute to rater’s ability to make an accurate judgment, and hence this study design does not evaluate these aspects of rater behavior that might facilitate reaching a judgment. However, our approach helps to promote consistency in the worm sample being assessed by the observers, and was likely essential to make gathering data from multiple raters feasible. By providing both a still image and movie, it is hoped that the range of views and behaviors available would be at least somewhat similar to what is available to a researcher making an assessment in the lab.

### Sources of inter-rater differences

Our findings suggest that differences in accuracy between the raters play a larger role than imprecision. In considering the threshold values, which characterize the differences in accuracy, a large number of comparisons may be made between the seven raters. However, in our study, we found that the distinction between the L1 and L2 and the L2 and dauer stages seemed to be most problematic as shown by the larger spread in the threshold values for the overall group (Table 5). Also contributing to these differences were specific large differences between pairs of raters (Fig 6). Our study was not focused on the origins of the differences in rater behavior, but given the finding that accuracy was more important than precision. Subsequent work that seeks to understand the visual cues involved in scoring would be of value. It is possible that the raters use the parameters of size, morphology of the gonad or other internal structures, and animal behavior to varying degrees in determining the developmental stage of the worms, and that the individual’s approach and weighting produces the observed differences. There may also be knowledge and experience differences that contribute as some of the raters in our group routinely staged animals in their research while others had other areas of expertise.

Regardless of the cause, the model provides specific information for how particular raters could adjust how they categorize worms, in order to agree more closely with the other raters in their lab. For example, rater 4 could review how to distinguish the younger larval stages from the older stages, due to his/her tendency to assign more worms to the L1 stage. Furthermore, rater 6 has a much higher threshold 4 value compared to the other raters, and rater 1 has a considerably lower threshold 1 value. This information would suggest that rater 6 tends to rate worms that have reached the L4 stage in earlier stages, and that rater 1 tends to rank L1 worms into higher categories. These raters could be mindful of these tendencies during subsequent scoring sessions.

Despite both computational and model complexity, this assessment is a worthwhile means of obtaining detailed and informative descriptions of inter-rater agreement, information that is not available by oversimplified and often misleading methods such as the computation of kappa coefficients. The results obtained can both provide confidence with regards to the success of the research team in accurately scoring the parameter of interest, and additionally the modeling approach provides insights into specific ways to improve the reliability of the raters.

## Materials and Methods

### Collecting worm rater data

To obtain a mixed stage population of worms containing dauer larvae as well as L1, L2, L3 and L4 stages, wild type N2 worms were grown at 20°C on nematode growth agar (NGA). This population was then supplemented with dauer larvae that were transferred from an *eak-4; tatn-1* double mutant stock, grown at 25°C. The *eak-4; tatn-1* double mutant will produce approximately 90% dauer larva when grown at 25°C [14]. Movies focused on small groups of worms within the mixed-stage NGA plate were obtained at 40X magnification using a Zeiss Stemi 2000 stereomicroscope with a color video camera attached. The I.C. capture 2.0 software was used to record the movies and still images. There were 9 separate 5 to 30 second movie files made, and each movie containing 3 to 10 worms that a rater would score. To ensure that all raters were scoring the same individual worms, the worms in these movies were each given a unique identifying number, and these were labeled in a separate image of a still from the first frame of each movie (Fig 2). The still images and movies were scored by seven individuals who had some amount of *C*. *elegans* research experience (Table 1). Before rating the worms, each rater was given a five-minute refresher tutorial by the author, explaining the qualifying features of each stage of development, and showing an example video containing each stage. Raters then were given a score-card, and for each movie file, were asked to identify the particular worm to be rated, and then play the video as needed in order to make a decision on the stage of development, and recording the result. This decision was typically reached after only a second or two of viewing the worm, and often a rating was reached after only viewing the still frame from the movie. These data were tabulated with a column for each rater. A total of seven raters were recruited for this study, and each rater was asked to judge the larval stage of 60 different worms. Hence each rater had 60 observations, with the larval stage of development coded by ordinal values one through five (S1 Table).

### Describing Agreement Using a Calibration Model with Thresholds

Although rating a worm for its stage of development depends on complex judgments, the resulting measurements are on a cruder scale compared to measurements of the worms’ length or weight. Length and weight are measured on a continuous, quantitative scale while determinations of the developmental stage are discrete measurements on an ordinal scale. Determining the agreement among measurements of length or weight made by different methods on the same set of items requires a measurement error model. The measurement error model describes how the common factor (the true values of the common items each method measures) influences each observed measurement. The measurement error model describes both the systematic differences (accuracy or relative bias) among the methods and the differences due to random error (imprecision). The relative bias between any two methods can then be removed by calibration. The same basic approach will work with ordinal ratings but the relative bias and random error are assumed to occur on an underlying continuous scale represented by continuous latent variables. The continuous latent variables then are translated to the observed discrete ordinal ratings via rater-specific threshold models.

The calibration model for three raters *i* = 1, 2, and 3 making measurements on a continuous scale for the *j* ^th^ worm, can be described by a set of three simultaneous equations:

$$X_{1j}\;=\;\alpha_{1}+\beta_{1}\mu_{j}+\epsilon_{1j}$$

$$X_{2j}\;=\;\alpha_{2}+\beta_{2}\mu_{j}+\epsilon_{2j}$$

$$X_{3j}\;=\;\alpha_{3}+\beta_{3}\mu_{j}+\epsilon_{3j}$$

Here, the true quantity is denoted by μ. The measurements are denoted by *X*. The systematic bias is described by α and β, and the random error by *∈*. These equations relate the measurements to the underlying, unknown true values. Because μ appears in all three equations, it is possible to relate *X*_1_ with *X*_2_, *X*_1_ with *X*_3_, and *X*_2_ with *X*_3_ –the μ drops out. That is, we can compare the raters even though the true values are never known. We can also describe the random error even though no item is measured more than once. (In effect, conditional on μ, we effectively have repeats for each method.)

A path diagram can be used to illustrate the calibration model, as shown in Fig 1. In this diagram, the quantity to be measured (the “true” value—a latent variable) is represented by the circle and the measurements by squares. The true value “causes” the observed measurements so there are single-sided arrows pointing from μ to each measurement. The *i* ^*th*^ rater distorts the true values and this distortion is quantified by α_*i*_ and *β*_*i*_. The double-sided arrow pointing to the circle for μ denotes the standard deviation σ for the true values. In a similar fashion, the standard deviations for each *∈*_*i*_ are denoted as σ_i_ next to the rectangle for each measurement *X*_i_. In this model, the scale bias parameter β_i_ and the random error parameter *∈*_*i*_.

When the measurements (*X*_i_) are ordinal, the calibration model can still be used but the ordinal categories (*k* = 1, …, 5) need to be modeled as outcomes based on an underlying (unobserved) normally distributed variable χ_i_ (mean of zero, standard deviation of one). We assume that the continuous true values μ are normally distributed with mean 0 and variance 1. It is convenient to constrain the unconditional normal distributions for each χ to a mean of zero and standard deviation of one. These constraints result in the following model:

$$\chi_{1j}\;=\;\rho_{1}\mu_{j}+\epsilon_{1j}$$

$$\chi_{2j}\;=\;\rho_{2}\mu_{j}+\epsilon_{2j}$$

$$\chi_{3j}\;=\;\rho_{3}\mu_{j}+\epsilon_{3j}$$

The intercepts vanish and the factor loadings (β) are equivalent to correlation coefficients and have been denoted by ρs. These parameters represent the correlation between each unobserved continuous rating χ with the unobserved true value μ. The constraints effectively remove any systematic error between the true values and the unobserved χ. Any systematic error is transferred to the *k* ^th^ threshold for subject *i t*_ik_. The area under the marginal distribution of χ_i_ below *t*_i1_ is the proportion of observations *X*_i_ for the first category labeled zero (0). Similarly, the area under the marginal distribution between *t*_i1_ and *t*_i2_ is the proportion of observations *X*_i_ for the second category labeled one (1), and so on. If there are *m* ordered categories, then there are *m-1* thresholds for each rater. Because the χ_i_ are latent variables, additional constraints are required to allow them to be identified. This requires that scale parameter ρ_i_ and the residual error *∈*_*i*_ standard deviation σ_i_ being related so that:

$$\sigma_{i}\;=\;\sqrt{1-\rho_{i}^{2}}$$

This constraint effectively means that ρ_i_ represents the inverse of the random error or precision (so that σ_i_ is the imprecision) while the thresholds *t*_ik_ represent the systematic error (bias).

This threshold model can be fitted using structural equation modeling software and the OpenMx R package was used. Full information maximum likelihood was used to estimate the model parameters. The one factor ordinal model with seven raters and four ordinal categories was adapted from code available from (http://openmx.psyc.virginia.edu/docs/OpenMx/latest/FactorAnalysisOrdinal_Matrix.html) [15, 16]. A detailed annotated version of the code used to produce the final results is shown in the supplemental material (S1 Text).

### Computational challenges in modeling worm rater data

There are definite benefits to fitting this type of model to the data, however, one challenge of this approach is the potential technical difficulty in achieving model convergence via the necessity of using an iterative solution. When iterative procedures are used, the software used to implement the procedure typically tries to assess whether or not convergence has been achieved. It should be remembered that this assessment is fallible. Just because no warning message was generated does not necessarily imply convergence and conversely, a warning message does not necessarily imply that convergence was not reached. In all cases, the output from iterative estimation procedure must be verified for convergence regardless of any warning messages or not. Iterative solutions typically require starting values for each parameter. The better the starting values, the more likely convergence will be achieved (and achieved in fewer iterations). Providing good starting values is akin to knowing the answer before having the answer. Many times determining good starting values is difficult so that random starting values are used. When using random starting values, it is customary to use a number of sets. When the common factor model (or similar) is used with continuous measurements, convergence is typically easier and faster to achieve. Threshold models needed for ordinal measurements are more challenging so that it is a good idea to run a larger number of sets of random starting values. The output of these random starts are collected and ordered by likelihood function value. Ideally, the outcome with the highest likelihood value should be the maximum likelihood solution. Often, with a large number of random sets the highest likelihood value will occur multiple times. Also, often very small differences in the likelihood value are connected with parameter estimates that are virtually identical, all leading to the conclusion that the maximum likelihood solutions has been achieved. Checking the parameter estimates to the observed data summaries (as we did) also helps confirm a reasonably correct solution. Additional techniques are to use software that implements the iterative procedure in an alternative fashion, and seeing that the solutions are either identical or very similar. We used all of these techniques to ensure the maximum likelihood solution was achieved.

## Supporting Information

S1 Table Primary data showing the scoring of individual worms by each rater. The numeric scores represent 1 for L1, 2 for L2, 3 for dauer, 4 for L3, and 5 for L4.
(DOCX)

S1 Text OpenMX code used for model fitting. (DOCX)
