# Supplementary material for: How Well Do Raters Agree on the Development Stage of Caenorhabditis elegans?
Source: PLoS One. 2015 Jul 14;10(7):e0132365. doi: 10.1371/journal.pone.0132365 (PMC4501796; doi:10.1371/journal.pone.0132365)
Supplement: S1 Table — The numeric scores represent 1 for L1, 2 for L2, 3 for dauer, 4 for L3, and 5 for L4. (DOCX) [file pone.0132365.s001.docx]

| **Worm ID** | **Rater 1** | **Rater 2** | **Rater 3** | **Rater 4** | **Rater 5** | **Rater 6** | **Rater 7** |
| --- | --- | --- | --- | --- | --- | --- | --- |
| **1** | 1 | 1 | 1 | 1 | 1 | 1 | 1 |
| **2** | 4 | 3 | 3 | 2 | 3 | 3 | 4 |
| **3** | 5 | 4 | 4 | 4 | 4 | 4 | 4 |
| **4** | 1 | 1 | 1 | 1 | 1 | 1 | 1 |
| **5** | 5 | 5 | 5 | 5 | 5 | 4 | 5 |
| **6** | 3 | 3 | 3 | 3 | 3 | 3 | 3 |
| **7** | 5 | 4 | 4 | 4 | 4 | 4 | 4 |
| **8** | 5 | 5 | 5 | 5 | 5 | 5 | 5 |
| **9** | 2 | 2 | 2 | 1 | 2 | 2 | 2 |
| **10** | 1 | 1 | 1 | 1 | 1 | 1 | 1 |
| **11** | 5 | 5 | 5 | 5 | 5 | 4 | 5 |
| **12** | 5 | 5 | 5 | 5 | 5 | 5 | 5 |
| **13** | 1 | 1 | 1 | 1 | 1 | 1 | 1 |
| **14** | 3 | 3 | 3 | 2 | 3 | 3 | 3 |
| **15** | 5 | 5 | 5 | 5 | 5 | 5 | 5 |
| **16** | 5 | 5 | 5 | 5 | 5 | 5 | 5 |
| **17** | 2 | 2 | 2 | 1 | 2 | 2 | 2 |
| **18** | 1 | 1 | 1 | 1 | 1 | 1 | 1 |
| **19** | 1 | 1 | 1 | 1 | 1 | 1 | 1 |
| **20** | 1 | 1 | 1 | 1 | 1 | 1 | 1 |
| **21** | 5 | 5 | 5 | 5 | 5 | 5 | 5 |
| **22** | 1 | 1 | 1 | 1 | 1 | 1 | 1 |
| **23** | 3 | 3 | 3 | 2 | 4 | 3 | 3 |
| **24** | 1 | 1 | 1 | 1 | 1 | 1 | 1 |
| **25** | 3 | 3 | 3 | 3 | 4 | 3 | 3 |
| **26** | 3 | 3 | 3 | 3 | 3 | 3 | 3 |
| **27** | 4 | 4 | 4 | 4 | 4 | 4 | 4 |
| **28** | 5 | 5 | 5 | 5 | 5 | 5 | 5 |
| **29** | 4 | 4 | 2 | 2 | 2 | 2 | 2 |
| **30** | 2 | 1 | 1 | 1 | 1 | 1 | 1 |
| **31** | 2 | 1 | 1 | 1 | 1 | 1 | 1 |
| **32** | 5 | 5 | 5 | 5 | 5 | 5 | 5 |
| **33** | 1 | 1 | 1 | 1 | 1 | 1 | 1 |
| **34** | 3 | 3 | 2 | 2 | 2 | 3 | 2 |
| **35** | 2 | 2 | 1 | 1 | 1 | 2 | 1 |
| **36** | 2 | 2 | 1 | 1 | 1 | 1 | 2 |
| **37** | 5 | 5 | 5 | 5 | 4 | 5 | 5 |
| **38** | 3 | 3 | 3 | 3 | 3 | 4 | 3 |
| **39** | 5 | 5 | 5 | 5 | 5 | 5 | 5 |
| **40** | 2 | 1 | 2 | 1 | 1 | 1 | 2 |
| **41** | 3 | 2 | 2 |  | 2 | 2 | 4 |
| **42** | 3 | 3 | 3 | 2 | 3 | 3 | 3 |
| **43** | 1 | 1 | 1 | 1 | 1 | 1 | 2 |
| **44** | 2 | 1 | 1 | 1 | 1 | 1 | 1 |
| **45** | 1 | 1 | 1 | 1 | 1 | 1 | 1 |
| **46** | 5 | 4 | 4 | 5 | 5 | 5 | 5 |
| **47** | 3 | 3 | 2 | 4 | 3 | 3 | 3 |
| **48** | 3 | 3 | 3 | 3 | 4 | 3 | 3 |
| **49** | 4 | 2 | 2 | 2 | 3 | 4 | 4 |
| **50** | 5 | 5 | 5 | 5 | 5 | 5 | 5 |
| **51** | 2 | 2 | 2 | 2 | 2 | 2 | 2 |
| **52** | 4 | 4 | 4 | 4 | 4 | 4 | 4 |
| **53** | 5 | 5 | 5 | 5 | 5 | 4 | 5 |
| **54** | 2 | 2 | 2 | 1 | 2 | 2 | 2 |
| **55** | 5 | 5 | 5 | 5 | 5 | 4 | 5 |
| **56** | 5 | 5 | 5 | 5 | 5 | 4 | 5 |
| **57** | 4 | 5 | 5 | 5 | 5 | 4 | 5 |
| **58** | 3 | 3 | 4 | 3 | 4 | 3 | 4 |
| **59** | 2 | 3 | 4 | 2 | 3 | 2 | 2 |
| **60** | 2 | 3 | 2 | 2 | 2 | 2 | 2 |
