# Supplementary material for: How Well Do Raters Agree on the Development Stage of Caenorhabditis elegans?
Source: PLoS One. 2015 Jul 14;10(7):e0132365. doi: 10.1371/journal.pone.0132365 (PMC4501796; doi:10.1371/journal.pone.0132365)
Supplement: S1 Text — (DOCX) [file pone.0132365.s002.docx]

**Open mx code: One factor model**

Annotated R code (Ordinal one common factor model, 7 raters, 5 categories)

(adapted from openMx documentation: http://openmx.psyc.virginia.edu/docs/OpenMx/latest/FactorAnalysisOrdinal_Matrix.html)

1) Read in the raw data (7 raters, 5 rating categories indicated with values 1, 2, 3, 4 and 5)

data_raw<-read.csv("/Volumes/NO NAME/thesis biostats/Movie_4_5categories.csv", sep=",",header=TRUE)

#remove worm_no

data_raters<-data_raw[,c("AAF", "SAK", "DAH", "UMA", "HNW", "ALF", "AGS")]

2) Convert the numerical values to ordered factors using the MxFactor command

ordinalData <- mxFactor(as.data.frame(data_raters),levels=c(1:(nThresholds+1)))

3) Label the number of raters (nVariables), number of latent factors (nFactors), and number of category thresholds (number of categories minus one, nThresholds), and rater names (raterNames) for the model.

nVariables<-7

nFactors<-1

nThresholds<-4

raterNames <- c("AAF", "SAK", "DAH", "UMA", "HNW", "ALF", "AGS")

4) To specify the model, first state a name for the model. Then generate a matrix to estimate the factor loadings, which has a row for each rater, and a single column (for the one factor). The Free=TRUE statement means that all the values will be freely estimated, and the lbound and ubound commands set the upper and lower limits of the factor loadings, which are standardized as a result of the common factor variance being fixed to one. The starting values for this matrix are given by the values= statement, which is set arbitrarily. This starting value may be adjusted if the program fails to converge on a minimum -2 log likelihood.

oneFactorThresholdModel <- mxModel("oneFactorThresholdModel",

mxMatrix(

type="Full",

nrow=nVariables,

ncol=nFactors,

free=TRUE,

values=0.96,

lbound=-.999,

ubound=.999,

name="facLoadings"

),

5) To constrain the variances of the observed variables to one, a matrix consisting of one column for each rater, with a value of one. This is subtracted from the factor loadings squared. The squared factor loadings are obtained by multiplying the facLoadings matrix by the transposed facLoadings matrix, and applying the diag2vec function to this result.

mxMatrix(

type="Unit",

nrow=nVariables,

ncol=1,

name="vectorofOnes"

),

mxAlgebra(

expression=vectorofOnes - (diag2vec(facLoadings %*% t(facLoadings))) ,

name="resVariances"

),

6) The expected covariances are obtained by adding each of the residual variances to the square of the factor loadings.

mxAlgebra(

expression=facLoadings %*% t(facLoadings) + vec2diag(resVariances),

name="expCovariances"

),

7) For the ordinal model, it is assumed that there is a latent distribution for each rater, with threshold values along this distribution that designate different categories. In this model, the latent distribution for each rater is constrained to the standard normal. Hence, the means for each rater are constricted to zero, while the threshold values are freely estimated. In the following statement, a zero vector is created, with columns for each rater.

mxMatrix(

type="Zero",

nrow=1,

ncol=nVariables,

name="expMeans"

),

8) To estimate the threshold values for each rater, first a matrix containing threshold deviations is created, with a row for each threshold, and a column for each rater. These values are all freely estimated. The lower bounds for each estimate are set as minus infinity for the first threshold, and then 0.01 for each subsequent threshold deviation. Again, the starting values in the matrix following the values= statement may be adjusted if the model fails to converge.

mxMatrix(

type="Full",

nrow=nThresholds,

ncol=nVariables,

free=TRUE,

values=c(-1, 0.5, 0.5, 0.5),

lbound=rep( c(-Inf,rep(.01,(nThresholds-1))) , nVariables),

dimnames=list(c(), raterNames),

name="thresholdDeviations"

),

9) These threshold deviations are converted to the expected thresholds by multiplying the thresholdDeviations matrix by a matrix with a lower half of ones.

mxMatrix(

type="Lower",

nrow=nThresholds,

ncol=nThresholds,

free=FALSE,

values=1,

name="unitLower"

),

mxAlgebra(

expression=unitLower %*% thresholdDeviations,

name="expThresholds"

),

10) The standard deviations are obtained by taking the square root of the variances. Additionally, likelihood based confidence intervals may be estimated for the threshold deviations.

mxAlgebra(sqrt(resVariances),"resSDs")

mxCI(c(“thresholdDeviations”)),

mxCI(c(“facLoadings”))

)

11) Finally, the model is fit, using the mxRun command, and the output and model summary are obtained with the @output and summary commands.

oneFactorFit <- mxRun(oneFactorThresholdModel, intervals=TRUE)

oneFactorFit@output

summary(oneFactorFit)

**Open MX Code: Serial Running of the Model with Random Starting Values to Ensure Convergence**

Due to the complexity of this model (having seven raters and five categories), and possibly because there were only 60 observations, there was a certain degree of computational challenge in achieving a minimum -2 log likelihood. The parameter estimates given by openMx came with a “code RED” warning message that stated the minimum -2LL may not have been achieved. In order to gain more confidence that the best parameter estimates were reached for this model, it was necessary to run the code using different starting values for the parameter estimates. This process was repeated over 100 times using slightly altered starting values for each run, and the parameter estimates from the model giving the lowest -2LL value were used in this final report. For this report, the results shown were generated from manually entering different starting values, and running the program many times. The best results from these attempts had a -2LL of 825.841. However, a code was also generated that automates the process of repeating the model with different starting values for each run, as shown in the supplemental material. In this code, the starting values for the parameter estimates were chosen at random from a uniform distribution, with maximum and minimum possible values specified. This code would enable repeating the model several hundred or thousands of times with relative ease, and potentially remove the error message, for future assessments.

1) After the data is read-in, the number of thresholds and raters is stated, and the data is converted to ordinal data, the model is built as before.

m1 <- mxModel("m1",

mxMatrix(

type="Full",

nrow=nVariables,

ncol=nFactors,

free=TRUE,

values=0.96,

lbound=-.999,

ubound=.999,

name="facLoadings"

),

mxMatrix(

type="Unit",

nrow=nVariables,

ncol=1,

name="vectorofOnes"

),

mxAlgebra(

expression=vectorofOnes - (diag2vec(facLoadings %*% t(facLoadings))) ,

name="resVariances"

),

mxAlgebra(

expression=facLoadings %*% t(facLoadings) + vec2diag(resVariances),

name="expCovariances"

),

mxMatrix(

type="Zero",

nrow=1,

ncol=nVariables,

name="expMeans"

),

mxMatrix(

type="Full",

nrow=nThresholds,

ncol=nVariables,

free=TRUE,

values=c(-1, 0.5, 0.5, 0.5),

lbound=rep( c(-Inf,rep(.01,(nThresholds-1))) , nVariables),

dimnames=list(c(), raterNames),

name="thresholdDeviations"

),

mxMatrix(

type="Lower",

nrow=nThresholds,

ncol=nThresholds,

free=FALSE,

values=1,

name="unitLower"

),

mxAlgebra(

expression=unitLower %*% thresholdDeviations,

name="expThresholds"

),

mxData(

observed=ordinalData,

type='raw'

),

mxFIMLObjective(

covariance="expCovariances",

means="expMeans",

dimnames=raterNames,

thresholds="expThresholds"

), mxAlgebra(sqrt(resVariances), "resSDs")

)

2) The following statements are required for fitting the model, and running it multiple times, with different starting values

3) First, the number of trials is stated:

trials <- 1000

4) Then the starting values for the parameter estimates are set up. These are set up as random values along the uniform distribution for each parameter estimate. For the factor loadings, the starting values are given between 0.8 and 0.99. For the threshold deviations, the first estimate is between -3 and 0, and the next three deviations fall between 0.1 and 0.5.

parNames <- names(omxGetParameters(m1))

input <- matrix(NA,trials,length(parNames))

dimnames(input) <- list(1:trials,parNames)

output <- matrix(NA,trials,length(parNames))

dimnames(output) <- list(1:trials,parNames)

fit <- matrix(NA,trials,4)

dimnames(fit) <- list(c(1:trials),c("Minus2LL","Status","Iterations","time"))

# Factor loadings

input[,"m1.facLoadings[1,1]"] <- runif(trials,0.8,0.99)

input[,"m1.facLoadings[2,1]"] <- runif(trials,0.8,0.99)

input[,"m1.facLoadings[3,1]"] <- runif(trials,0.8,0.99)

input[,"m1.facLoadings[4,1]"] <- runif(trials,0.8,0.99)

input[,"m1.facLoadings[5,1]"] <- runif(trials,0.8,0.99)

input[,"m1.facLoadings[6,1]"] <- runif(trials,0.8,0.99)

input[,"m1.facLoadings[7,1]"] <- runif(trials,0.8,0.99)

# Thresholds for rater 1

input[,"m1.thresholdDeviations[1,1]"] <- runif(trials,-3,0)

input[,"m1.thresholdDeviations[2,1]"] <- runif(trials,0.1,0.5)

input[,"m1.thresholdDeviations[3,1]"] <- runif(trials,0.1,0.5)

input[,"m1.thresholdDeviations[4,1]"] <- runif(trials,0.1,0.5)

# Thresholds for rater 2

input[,"m1.thresholdDeviations[1,2]"] <- runif(trials,-3,0)

input[,"m1.thresholdDeviations[2,2]"] <- runif(trials,0.1,0.5)

input[,"m1.thresholdDeviations[3,2]"] <- runif(trials,0.1,0.5)

input[,"m1.thresholdDeviations[4,2]"] <- runif(trials,0.1,0.5)

# Thresholds for rater 3

input[,"m1.thresholdDeviations[1,3]"] <- runif(trials,-3,0)

input[,"m1.thresholdDeviations[2,3]"] <- runif(trials,0.1,0.5)

input[,"m1.thresholdDeviations[3,3]"] <- runif(trials,0.1,0.5)

input[,"m1.thresholdDeviations[4,3]"] <- runif(trials,0.1,0.5)

# Thresholds for rater 4

input[,"m1.thresholdDeviations[1,4]"] <- runif(trials,-3,0)

input[,"m1.thresholdDeviations[2,4]"] <- runif(trials,0.1,0.5)

input[,"m1.thresholdDeviations[3,4]"] <- runif(trials,0.1,0.5)

input[,"m1.thresholdDeviations[4,4]"] <- runif(trials,0.1,0.5)

# Thresholds for rater 5

input[,"m1.thresholdDeviations[1,5]"] <- runif(trials,-3,0)

input[,"m1.thresholdDeviations[2,5]"] <- runif(trials,0.1,0.5)

input[,"m1.thresholdDeviations[3,5]"] <- runif(trials,0.1,0.5)

input[,"m1.thresholdDeviations[4,5]"] <- runif(trials,0.1,0.5)

# Thresholds for rater 6

input[,"m1.thresholdDeviations[1,6]"] <- runif(trials,-3,0)

input[,"m1.thresholdDeviations[2,6]"] <- runif(trials,0.1,0.5)

input[,"m1.thresholdDeviations[3,6]"] <- runif(trials,0.1,0.5)

input[,"m1.thresholdDeviations[4,6]"] <- runif(trials,0.1,0.5)

# Thresholds for rater 7

input[,"m1.thresholdDeviations[1,7]"] <- runif(trials,-3,0)

input[,"m1.thresholdDeviations[2,7]"] <- runif(trials,0.1,0.5)

input[,"m1.thresholdDeviations[3,7]"] <- runif(trials,0.1,0.5)

input[,"m1.thresholdDeviations[4,7]"] <- runif(trials,0.1,0.5)

5) A loop is set up that will fit the model the number of times specified in the trials statement, and will give starting values for the parameter estimates according to the input statements above.

# Loop to fit models

for(i in 1:trials) {

temp1 <- omxSetParameters(m1,

labels=parNames,

values=input[i,]

)

temp1@name <- paste("Starting Values Set",i)

temp2 <- mxRun(temp1,unsafe=TRUE,suppressWarnings=TRUE)

output[i,] <- omxGetParameters(temp2)

fit[i,] <- c(

temp2@output$Minus2LogLikelihood,

temp2@output$status[[1]],

temp2@output$iterations,

temp2@output$wallTime

)

print(output[i,])

print(fit[i,])

print(head(table(round(fit[,1],3),fit[,2])))

}

save.image(file="/Users/AAF/Desktop/AAFresults2.RData")
